# Identification of Candidate Expansin Genes Associated with Seed Weight in Pomegranate (*Punica granatum* L.)

**DOI:** 10.3390/genes15020212

**Published:** 2024-02-06

**Authors:** Chunyan Liu, Haoyu Zhao, Jiyu Li, Zhen Cao, Bo Deng, Xin Liu, Gaihua Qin

**Affiliations:** 1 Key Laboratory of Horticultural Crop Germplasma Innovation and Utilisation (Co-Construction by Ministry and Province), Institute of Horticulture, Anhui Academy of Agricultural Sciences, Hefei 230031, China; liucy17@163.com (C.L.); 18298157188@163.com (H.Z.); lijiyugx@163.com (J.L.); hazelcz@163.com (Z.C.); l498564811@163.com (X.L.); 2 Key Laboratory of Genetic Improvement and Eco-Physiology of Horticultural Crops, Institute of Horticulture, Anhui Academy of Agricultural Sciences, Hefei 230031, China; 3 School of Forestry and Landscape Architecture, Anhui Agricultural University, Hefei 230036, China; bdeng2008@sohu.com

**Keywords:** pomegranate, expansin, seed weight, marker, association analysis

## Abstract

Seed weight is an important target trait in pomegranate breeding and culture. Expansins act by loosening plant cell walls and cellulosic materials, permitting turgor-driven cell enlargement. However, the role of expansin genes (*EXPs*) in pomegranate seed weight remains elusive. A total of 29 *PgrEXPs* were identified in the ‘Dabenzi’ genome. These genes were classified into four subfamilies and 14 subgroups, including 22 *PgrEXPAs*, 5 *PgrEXPBs*, 1 *PgrEXPLA*, and 1 *PgrEXPLB*. Transcriptome analysis of *PgrEXPs* in different tissues (root, leaf, flower, peel, and seed testa) in ‘Dabenzi’, and the seed testa of the hard-seeded pomegranate cultivar ‘Dabenzi’ and soft-seeded cultivar ‘Tunisia’ at three development stages showed that three *PgrEXPs* (*PgrEXPA11*, *PgrEXPA22*, *PgrEXPA6*) were highly expressed throughout seed development, especially in the sarcotesta. SNP/Indel markers of these *PgrEXPs* were developed and used to genotype 101 pomegranate accessions. The association of polymorphic *PgrEXPs* with seed weight-related traits (100-seed weight, 100-kernel weight, 100-sarcotesta weight, and the percentage of 100-sarcotesta to 100-seed weight) were analyzed. *PgrEXP22* was significantly associated with 100-seed weight and 100-sarcotesta weight and is a likely candidate for regulating seed weight and sarcotesta development in particular. This study provides an effective tool for the genetic improvement of seed weight in pomegranate breeding programs.

## 1. Introduction

Pomegranate, *Punica granatum* L., is an ancient crop that originated from Central Asia and is now widely grown in subtropical and tropical areas [1]. Pomegranate produces fruit that is valued for its juice-containing seeds and health benefits. The edible proportion of the fruit depends largely on the size of the fleshy outer testa; therefore, the size of the seeds, and that of the juicy outer testa in particular, has become an important fruit characteristic [2]. Pomegranates exhibit considerable phenotypic diversity in seed size among different genotypes, and seed size, juice acidity, and pH are the most useful factors for the genetic characterization of pomegranate germplasm banks [3]. Additionally, seed size is affected by the rate of supplied nitrogen [4] and pollination methods [5].

Pomegranate seeds can be defined as a reproductive structure that has a fleshy outer testa called the sarcotesta and an internal sclerotic testa called the mesotesta [6,7]. The juicy edible layer has a single layer of translucent and pulpy columnar cells, which elongate to a very large extent in a radial direction [7]. Such cell enlargement is achieved economically by filling the cell with a large central vacuole containing water and solutes. The physical control of this process resides in the ability of the cell wall to undergo turgor-driven expansion [8]. Expansins are ‘factors that loosen the cell wall’, acting by disrupting noncovalent associations between cellulose and matrix polysaccharides in the plant cell wall, allowing the polymers to slip relative to one another and thereby permitting turgor-driven cell enlargement [8]. Expansins are typically 250–275 amino acids long and are made up of two domains preceded by a signal peptide. Domain I is a six-stranded double-psi β-barrel (DPBB), which comprises more than 120 amino acids with a His-Phe-Asp (HFD) motif and conserved cysteine residues (N-terminal); and domain II is homologous with group 2 grass pollen allergens [9] and was recently classified as a family-63 carbohydrate-binding module (CBM63). Plant expansin genes (*EXPs*) are divided into four families: α-expansin (EXPA), β-expansin (EXPB), expansin-like A (EXLA), and expansin-like B (EXLB), with α-expansin and β-expansin more clearly associated with cell expansion and growth [10,11].

Expansins are plant cell-wall-loosening proteins involved in cell enlargement and in a variety of other developmental processes associated with cell wall modification, including fruit firmness and softening [12], root development and growth [13], leaf initiation, and leaf growth [14]. Moreover, EXPA genes affect seed development and seed size. The over-expression of an α-expansin gene (*TaExpA6*) in early developing wheat seeds led to a significant increase in grain size without a negative effect on grain number, resulting in a yield boost under field conditions [15]. The over-expression of a sweet potato β-expansin gene (*IbEXP1*) resulted in plants with thicker siliques and larger seeds [16]. Expansins in growing ovaries and grains of sunflowers showed tissue specificity and were associated with final grain weight [17]. These results show that *EXPs* play an important role in the seed expansion process; however, the role of *EXPs* in pomegranate seed development is currently poorly understood.

Three draft genome sequences of pomegranates, including ‘Dabenzi’, with an assembled size of 328 Mb [18]; ‘Tunisia’, with an assembled size of 320 Mb [19]; and ‘Taishanhong’, with an assembled size of 274 Mb [20], are currently available, offering an essential molecular basis for pomegranate gene family analysis. In this study, we report the identification of *PgrEXPs* in the ‘Dabenzi’ reference genome and examine expression profiles during pomegranate seed development. SNP/Indel markers for highly expressed *PgrEXPs* were developed to investigate their association with seed weight. This study not only aids our understanding of the effect of *PgrEXPs* on seed weight, but it will also be useful for the genetic improvement of seed weight in breeding programs.

## 2. Materials and Methods

### 2.1. Plant Materials

The 101 pomegranate accessions (Supplementary File S1) used in this study were planted at the Gangji Eco-Agricultural Demonstration site, Anhui Academy of Agricultural Sciences, Hefei, Anhui, China. Young leaves used for DNA extraction were immediately frozen at −80 °C until use. Nine ripe fruits were randomly collected from each pomegranate accession in 2020.

### 2.2. Measurements of Seed Weight Traits

After extracting the seeds by hand, 100 of them were randomly selected and weighed. The remaining seeds were squeezed by hand to obtain the kernels, and 100 kernels were randomly selected for weighing. The following formula was used: 100-sarcotesta weight = 100-seed weight − 100-kernel weight. PSW is defined as the percentage of 100-sarcotesta to 100-seed weight: $PSW=\frac{100-sarcotesta\;weight}{100-seed\;weight}\times 1$00%. Each accession had three replicates. The column chart distribution of seed weight in 101 pomegranate accessions was generated using Origin.

### 2.3. Identification of the PgrEXPs and Phylogenetic Tree Construction

The whole-genome and protein sequences of ‘Dabenzi’ were used in this study [18]. The Arabidopsis expansin protein sequences were downloaded from The Arabidopsis Information Resource (http://www.arabidopsis.org/, accessed on 29 January 2024). Expansin protein sequences of Chinese jujube (*Ziziphus jujuba* Mill.) and eucalyptus *(Eucalyptus grandis*) were collected from the NCBI database (https://www.ncbi.nlm.nih.gov/, accessed on 29 January 2024). Firstly, the Arabidopsis expansin protein sequences were used as queries to perform tblastn against the pomegranate genome sequence database with a 1 × 10^−5^ cut-off. Then, all protein sequences of the putative EXPs were scanned for the EXP conserved domains using InterProScan (http://www.ebi.ac.uk/interpro/, accessed on 29 January 2024). The sequences lacking expansin domains were removed, the remaining sequences were combined as candidate genes, and then CDD (http://www.ncbi.nlm.nih.gov/Structure/cdd/wrpsb.cgi, accessed on 29 January 2024) and SMART (http://smart.embl-heidelberg.de/, accessed on 29 January 2024) were used to further verify the candidate protein sequences.

The phylogenetic tree was constructed using MEGA software (http://www.megasoftware.net/, accessed on 29 January 2024) [21] using the neighbor-joining (NJ) method with 1000 bootstrap replicates. 

### 2.4. Protein Properties, Chromosomal Location, and Gene Structure Analysis

The peptide length, molecular weight (MW), and isoelectric point (pI) of the PgrEXPs were calculated using the online ExPasy program (https://www.expasy.org/, accessed on 29 January 2024). Subcellular localization was predicted by WoLF PSORT (https://www.genscript.com/wolf-psort.html, accessed on 29 January 2024).

The specific chromosomal location of each PgrEXP was obtained from the pomegranate genome database. Two or more adjacent genes within a region of 200 kb located on a chromosome were defined as tandem duplications, and homologous relationships were drawn using the Circos software [22]. The gene structure information was analyzed using the GSDS website (http://gsds.cbi.pku.edu.cn/, accessed on 29 January 2024) [23]. The conserved motifs of the PgrEXPs were identified using the MEME website (http://meme-suite.org/) [24], with a maximum of 10 motifs. The structures of the genes and conserved motifs were generated using TBtools [25]. 

### 2.5. Transcriptome Analysis of the PgrEXP Genes

The abundances of *PgrEXP* transcripts in different tissues of ‘Dabenzi’ and in the inner (mesotesta) and outer (sarcotesta) seed coats of the hard-seeded cultivar ‘Dabenzi’ and soft-seeded cultivar ‘Tunisia’ were obtained from the NCBI sequence read archive (SRA) with accession codes SRP100581 and SRP212814 [18]. Transcriptional abundances were estimated using the fragments per kilobase of exon per million mapped reads (FPKM) values and illustrated with a heat map based on the log_2_ FRKM transformation value generated by TBtools. 

### 2.6. DNA Extraction, Primer Design, PCR Sequencing, and Gene-Based Association Analysis

Genomic DNA was extracted from young leaves using a modified CTAB method [26]. Primers were designed by Primer Premier Version 5.0 (Premier Biosoft International, PaloAlto, CA, USA), and primer details are provided in Supplementary File S2. The PCR procedure took place at 95 °C for 3 min, followed by 35 cycles of 95 °C for 30 s, annealing (55–60 °C) for 30 s, and an extension of 72 °C for 30 s, with a final extension of 72 °C for 10 min. The PCR products were separated by electrophoresis in agarose, and PCR products were sent to General Biosystems (Anhui) Corporation Limited for sequencing. The sequencing results were aligned and filtered using the Bioedit software to genotype the mutation sites. The association between the genotypes of the mutation site and growth traits was analyzed using the one-way analysis of variance (ANOVA) in SPSS 20 software, with a significance level of *p* < 0.05. 

## 3. Results

### 3.1. The PgrEXPs in the Pomegranate Genome

**Figure 1:**
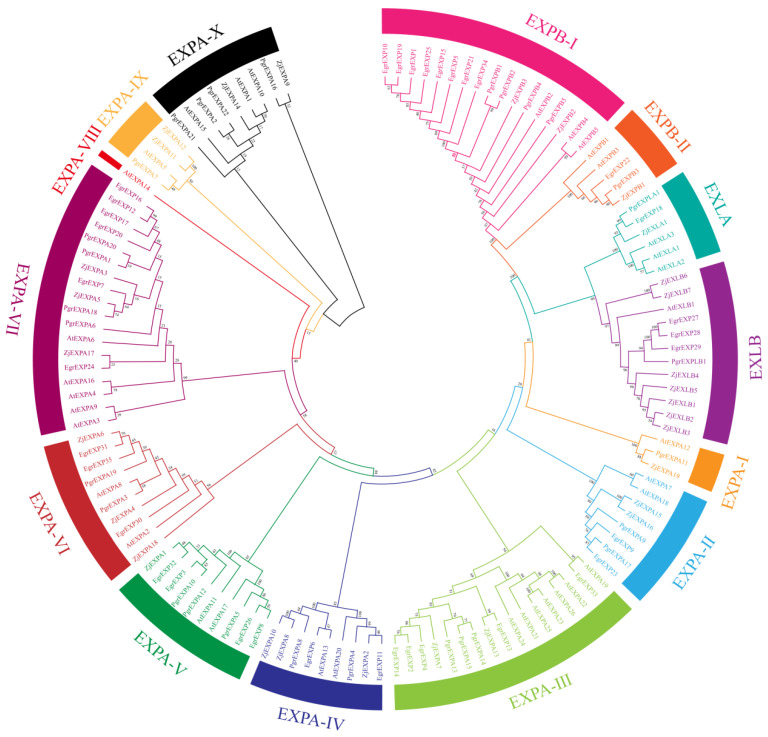
Phylogenetic analysis of the expansin genes in *Arabidopsis thaliana* (*AtEXP*), *Z. jujuba*. (*ZjEXP*), *E. grandis* (*EgrEXP*), *P. granatum* (*PgrEXP*). The subgroups are marked by different color bars.

A total of 29 *PgrEXPs* were identified in the genome of *P. granatum cv.* ‘*Dabenzi*’. Each gene was named on the basis of family identity and chromosomal position according to nomenclature guidelines [27]. The phylogenetic tree showed that the expansin gene family is divided into four subfamilies: EXPA, EXPB, EXLA, and EXLB. The EXPA subfamily was the largest group, with 22 members, and the EXPB subfamily contained 5 members (Figure 1). The EXLA and EXLB subfamilies contained one member each. The four expansin gene subfamilies were further divided into 14 subgroups according to the grouping rules used for the *Arabidopsis* and jujube gene families [9,28].

**Figure 2:**
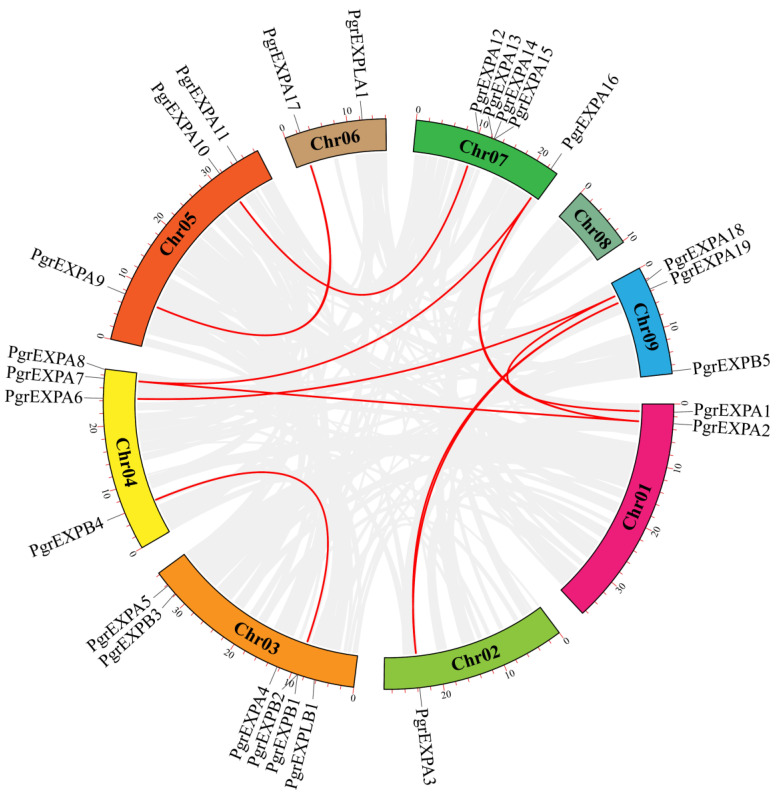
Collinearity analysis of expansin genes in pomegranate. Red lines indicate the collinearity of expansin genes among chromosomes in pomegranate; gray lines in the background indicate all the collinear blocks among different chromosomes.

The chromosomal distribution map showed that the *PgrEXPs* were unevenly distributed on each chromosome. Of the 29 *PgrEXPs*, 26 were located on eight chromosomes (ranging from 1 to 6 on each), while 3 *PgrEXPs* (*PgrEXPA20*, *PgrEXPA21*, *PgrEXPA22*) were mapped on unplaced genomic scaffolds. Duplicated genes via tandem duplications were detected on chromosome 3, containing *PgrEXPB1* and *PgrEXPB2*, and chromosome 7, containing *PgrEXPA14* and *PgrEXPA15*. Collinearity relationship analysis revealed that a total of nine pairs of duplication genes were detected in the *PgrEXPs* (Figure 2). These results suggest that tandem duplication and fragment duplication are likely to have been the driving force in *PgrEXP* evolution. 

**Figure 3:**
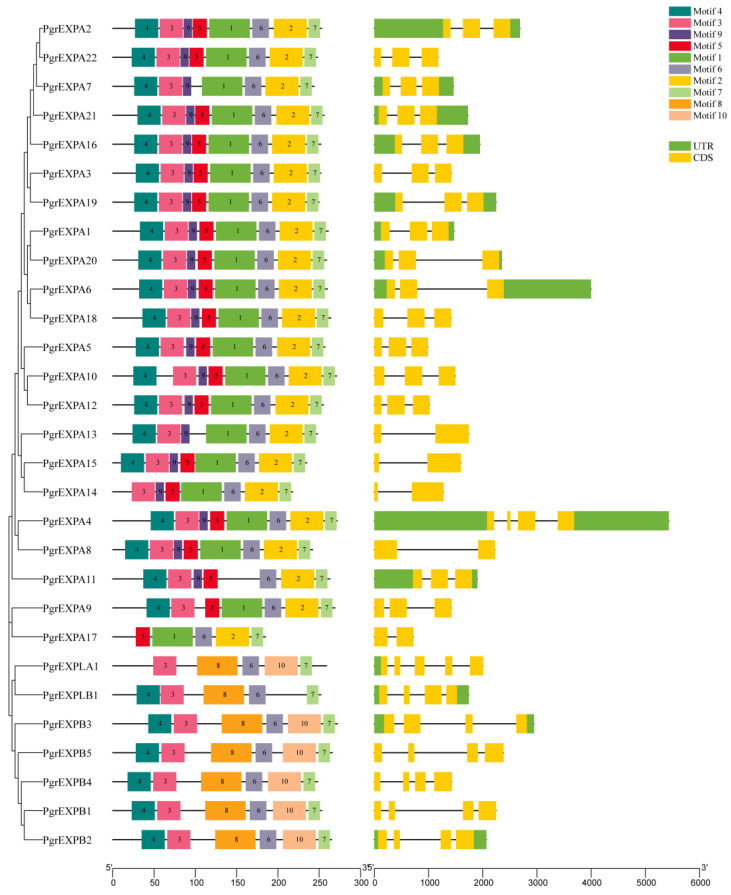
Protein domain architecture and gene structure of PgrEXPs. The Leaf figure shows the conserved motif composition of PgrEXP proteins. The motifs (numbered 1–10) are displayed in different colored boxes. The right figure shows the exon–intron structure of PgrEXPs. The green boxes indicate untranslated regions (UTR), yellow boxes indicate exons, and black lines indicate introns.

The *PgrEXPs* encoded proteins ranging from 185 (*PgrEXPA17*) to 272 (*PgrEXPB3*, *PgEXPA4*) amino acids (aas) in length, with an average length of 252 aas, and had predicted molecular weights of 20.50–29.86 kDa (Supplementary File S3). Ten distinct motifs were detected in *PgrEXPs,* and the number of motifs in *PgrEXP* family members ranged from five to eight (Figure 3). The multiple sequence alignment results of 29 proteins showed that they had similar sequence characteristics: the majority of them consisted of a signal peptide and conserved domain I and domain II (Supplementary File S4), which was consistent with the findings of a previous study [9]. Motifs 1, 3, 5, and 9 encoded the well-conserved N-terminal domain 1, and motifs 2 and 6 encoded the well-conserved C-terminal domain 2. At least two of the six motifs were shared among the pomegranate expansin genes. In addition, all members contained motifs 6 and 7, indicating that these motifs are closely related to the biological function and sequence characteristics of pomegranate. An exon–intron analysis indicated that the number of exons contained in each PgrEXP ranged from two to five. In general, members within each subgroup or subfamily exhibited similar gene structures in terms of exon length and number (Figure 3). 

### 3.2. Expression Patterns of PgrEXPs Revealed by Transcriptome Analysis

**Figure 4:**
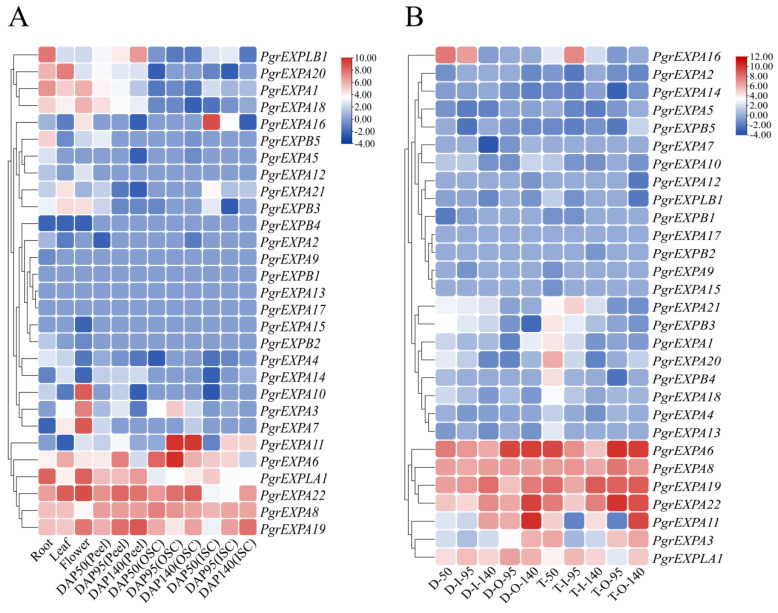
Expression profiles of the *PgrEXPs* in different tissues and varieties of pomegranate. (**A**): The transcript data of *PgrEXPs* in the root, flowers, and leaf as well as the peel, sarcotesta, and mesotesta at three development stages (50, 95, and 140 days after pollination) were processed with log2 normalization based on FPKM values. “OSC” = sarcotesta, “ISC” = mesotesta. (**B**): The transcript data of *PgrEXPs* in the mesotesta and sarcotesta of hard-seeded cultivar ‘Dabenzi’ and soft-seeded cultivar ‘Tunisia’ at three development stages (50, 95, and 140 days after pollination) were processed with log2 normalization based on FPKM values. “D” = ‘Dabenzi’; “T” = ‘Tunisia’, “O” = sarcotesta, and “I” = mesotesta. The subsequent number represents the days after pollination.

To analyze the expression profiles of individual *PgrEXPs*, the available transcriptome data published in the NCBI (accession number SRP 100581, PRJNA548841) were used to construct a hierarchical clustering heat map showing the expression levels of *PgrEXPs* in different tissues (Figure 4A). According to the hierarchical clustering, the expression patterns of *PgrEXPs* were divided into five groups. The genes (*PgrEXPLB1*, *PgrEXPA20*, *PgrEXPA1*, *PgrEXPA18*) in group Ⅰ exhibited extremely high expression patterns in the roots, leaves, flowers, and peel. The expression levels of all genes in group Ⅱ were extremely low in all tissues, except for *PgrEXP16*, which was more highly expressed in the mesotesta. All genes in group Ⅲ were either not expressed in any tissues or rarely expressed. *PgrEXPA3*, *PgrEXPA7*, and *PgrEXPA10* from group Ⅳ were exclusively expressed in flowers, indicating that these genes may play important functions in flower development. All genes from group Ⅴ except for *PgrEXP11* were highly expressed in all tissues. *PgrEXPA11* and *PgrEXPA6* were highly expressed in the seed testa, suggesting that these genes may be involved in seed testa development. 

Additionally, we analyzed the spatiotemporal expression patterns of all *PgrEXPs* in the seed testa of the hard-seeded pomegranate cultivar ‘Dabenzi’ and soft-seeded cultivar ‘Tunisia’ at three development stages (Figure 4B). The results showed that *PgrEXPA6* and *PgrEXPA22* were highly expressed in the seed testa of both cultivars and that the accumulation of these genes in the sarcotesta was significantly higher during the late stages compared to the early stages of seed development. Moreover, *PgrEXPA11* in particular was highly expressed in the sarcotesta during the mature stage of seed development. Hence, these three genes were assumed to be potential candidates related to sarcotesta development and were further evaluated to determine their genetic association with seed weight.

### 3.3. Association between PgrEXPs and Seed Weight in Pomegranate

**Figure 5:**
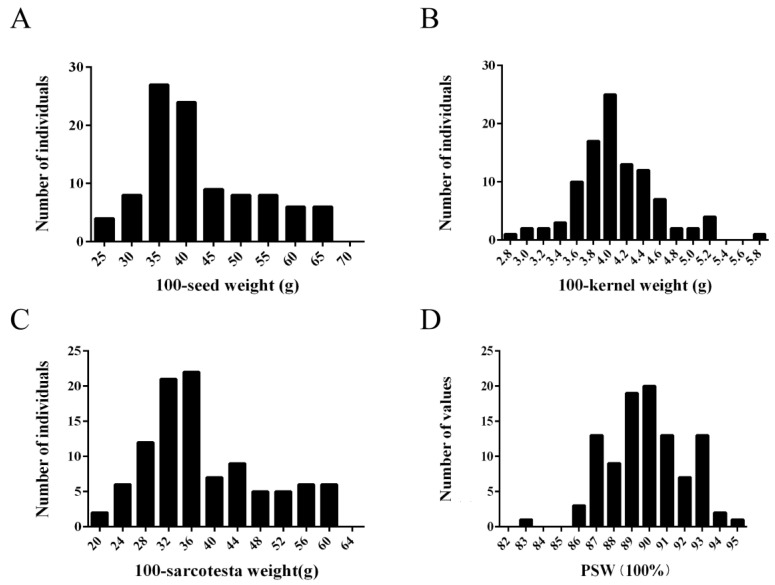
Distribution of seed weight-related parameters for mature fruits of pomegranate accessions. (**A**) 100-seed weight; (**B**) 100-Kernel weight; (**C**) 100-sarcotesta weight; (**D**) PSW.

One hundred and one pomegranate accessions were used to carry out the association analysis between *PgrEXPs* and seed weight. Seed weight-related traits, including 100-seed weight, 100-kernel weight, 100-sarcotesta weight, and PSW, showed a normal distribution (Figure 5). Overall, the pomegranate accessions showed great variation in seed weight, including 100-seed weight (25.42–65.95 g), 100-kernel weight (2.87–5.79 g), 100-sarcotesta weight (21.13–61.41 g), and PSW (82.89–94.76%), with averages of 42.31 g, 4.08 g, 38.23 g, and 89.93%, respectively, which suggests that they are suitable for investigating the genetic association of *PgrEXPs* with seed weight.

Twenty accessions were selected to screen the polymorphism loci in three genes (*PgrEXPA11*, *PgrEXPA22*, *PgrEXPA6*). No polymorphism loci were found in *PgrEXPA11,* and the genes (*PgrEXPA22*, *PgrEXPA6*) with polymorphism loci were further amplified in all of the 101 accessions. Sequence alignment showed three SNPs and one indel in *PgrEXPA22*: a T/C mutation at position 696 bp in the exon, a T/A mutation at position 1004 bp in the intron, a T/G mutation at position 1779 bp in the UTR, and a TGA insertion at position 1088 bp. Five SNPs were found in *PgrEXPA6*: an A/G mutation at position 1170 bp in the intron, a T/C mutation at position 1237 bp in the intron, a C/G mutation at position 1658 bp in the intron, and a C/G mutation at position 2538 bp in the exon (Table 1). 

The correlation between the different genotypes of the *PgrEXPs* at nine polymorphic loci and the seed weight-related traits of pomegranate was analyzed (Table 2). As a result, four loci in *PgrEXP22* were significantly associated with 100-seed weight and 100-sarcotesta weight, and one locus in *PgrEXP22* was associated with PSW. Three alleles, TT, CC, and TC, were identified at g.696T>C through direct sequencing of PCR products. Accessions with TT alleles had significantly higher 100-seed weight, 100-sarcotesta weight, and PSW compared to those with the CC and TC alleles (*p* < 0.05). There were no differences in 100-seed weight, 100-sarcotesta weight, and PSW between the CC and TC alleles. Similarly, all the accessions were divided into three alleles, AA, AT, and TT, at the g.1004T>A locus. Accessions with TT alleles had significantly higher 100-seed weight and 100-sarcotesta weight compared to those with AA alleles (*p* < 0.05), but no differences were found between TT and AT or between AT and AA. The g.1088_1089insTGA locus had three alleles, and the homozygous insertion site (INDEL+INDEL+) showed significantly higher 100-seed weight and 100-sarcotesta weight compared to sites with no TGA insertion (INDEL-INDEL-) (*p* < 0.05), but no differences were found between INDEL+INDEL+ and INDEL+INDEL- or between INDEL+INDEL- and INDEL-INDEL-. The g.1779T>G locus also had three alleles: TT, TG, and GG. The TT genotype showed significantly higher 100-seed weight and 100-sarcotesta weight compared to the GG genotype (*p* < 0.05), but no differences were found between TT and TG or between TG and GG. Collectively, the results demonstrated that the loci of TT at g.696T>C, TT at g.1004T>A, INDEL+INDEL+ at g.1088_1089insTGA, and TT at g.1779T>G had a significant positive effect on seed size and are potentially superior alleles for the improvement of seed yield in pomegranate. It is worth mentioning that all the loci in *PgrEXP22* had no effect on the 100-kernel weight. *PgrEXPA6* had no effect on the seed weight-related traits.

## 4. Discussion

Seed size is a key determinant of evolutionary fitness and is one of the most important components of seed yield. The size of a seed is governed by the coordinated growth of the embryo, endosperm, and maternal tissues like the ovule [29]. Various genetic pathways such as IKU, KLUH, ubiquitin proteasome, and BR-mediated pathways govern cell proliferation and expansion in the three seed compartments [30]. Seed weight, especially sarcotesta weight, which determines the edibility of fruits, has become an important characteristic of pomegranate. Expansins unlock the network of wall polysaccharides, permitting turgor-driven cell enlargement. Although preliminary analyses of the expansin gene family have been reported in pomegranate [31], little is known about the role that *EXPs* play in seed weight. In this study, we report the genetic association of *PgrEXPs* with the seed weight of pomegranates for the first time.

A total of 29 *PgrEXPs* were detected in the ‘Dabenzi’ pomegranate genome. The number of *PgrEXPs* in our study is inconsistent with a previous report that identified a total of 33 pomegranate *PgrEXPs*. This inconsistency is likely due to the fact that our analysis is based on the draft genome sequence of ‘Dabenzi’ [18], while the genome sequence of pomegranate cv was utilized in the previous report. Taishanhong was used in the study of Xu et al. [31]. Similar results were found in the SWEET gene, and the number of SWEET genes identified in the Taishanhong genome was greater than that identified in Dabenzi [32,33]. Although the number of PgrEXPs varied among cultivars and species, expansins in pomegranate and the known expansins of the other species were divided into four subfamilies based on phylogenetic analysis [9,28,31]. Gene duplication is one of the primary driving forces in the evolution of genomes and genetic systems in plants. In this study, tandem duplication and a pair of genes from fragmental replication were detected among the PgrEXPs. For example, a cluster of three genes, *PgrEXPA13*, *PgrEXPA14* and *PgrEXPA15*, was found on chromosome 7. Similarly, a cluster of two *PgrEXPs*, *PgrEXPB1* and *PgrEXPB2*, was found on chromosome 3. These results suggest that the tandem duplication of expansins probably occurred on chromosomes 3 and 7. In addition, it is worth noting that nine pairs of genes from fragmental replication were found. These findings are consistent with the expansins found in jujube [28] and cucumber [34]. 

A measurement of the seed weight of 101 pomegranate accessions revealed that the sarcotesta weight accounted for 89.95% of the seed weight on average, ranging from 82.89% to 94.76%. Correlation analysis revealed that 100-seed weight exhibits a significantly positive correlation with 100-sarcotesta weight and 100-kernel weight, particularly with the 100-sarcotesta weight (r = 0.999***), which confirms previous reports of seed weight being determined by the sarcotesta [35]. Moreover, the three traits of seed weight exhibited a continuous distribution. A study conducted by Harel-Beja also showed that the size of pomegranate seeds is a typical quantitative trait, which exhibited stability across different years. Six QTLs were identified for seed weight, but the identified QTLs were located in larger chromosomal segments, making it difficult to precisely identify QTLs or genes associated with seed weight [36]. Studies of associations within collections can yield a broader view of the number of loci involved in the control of complex traits. The released pomegranate genome sequences are crucial tools for the functional analysis of genes and associate gene function with important agronomic traits. Therefore, a candidate gene-based association study was used to identify the genes underlying the seed weight. 

The pulpy sarcotesta is composed of a single layer of radial cells, which elongates radially to many times its original length and becomes turgid during pomegranate seed development [7]. Cell volume increases as a result of water uptake, often into the cell vacuoles, and cell wall stress relaxation occurs, which results in the cell walls loosening [37]. Expansins have been shown to induce cell wall loosening to promote cell wall enlargement by disrupting the noncovalent bonds between cellulose microfibrils and xyloglucan [9]. In this study, an analysis of temporal transcript abundance patterns revealed the higher expression of seven genes during seed development. The transcripts of these PrgEXPs in the mesotesta changed less during seed development compared to the sarcotesta, as the changes in mesotesta volume were not as significant as those in the sarcotesta [7]. Furthermore, the transcripts of *PgrEXPA11*, *PgrEXPA22*, and *PgrEXPA6* were associated with the seed weight during seed development, implying their role in sarcotesta enlargement. Interestingly, *TaExpA6* ectopic expression increases grain size and weight in transgenic wheat [15]. Based on the phylogenetic tree, *PgrEXP 22* is closely related to *AtEXPA10*, which affects the sizes of leaves, stems, and flowers [38]. Therefore, *PgrEXPA11*, *PgrEXPA22*, and *PgrEXPA6* may regulate pomegranate seed weight by promoting cell expansion, and so these genes were used for further candidate gene association analysis.

Since Thornsberry et al. first utilized candidate gene association analysis in maize with 92 materials to study the gene *Dwarf8* in 2001 [39], the candidate gene-based association analysis method has been widely used in plants. In this study, an association analysis between the different genotypes of nine SNPs within the *PgrEXPs* and the seed weight traits in 101 pomegranate accessions was conducted. As a result, four loci in *PgrEXP22* were significantly associated with 100-seed weight and 100-sarcotesta weight; one locus in *PgrEXP22* was significantly associated with PSW, and no SNPs were associated with 100-kernel weight, indicating that the key genes involved in the expansion of the sarcotesta and mesotesta are different. Furthermore, g.696T>C is a polymorphism likely associated with 100-seed weight, 100-sarcotesta weight, and PSW, with the TT genotype having positive effects on these three traits. An SNP can cause a strong influence on genes and further lead to changes in phenotypes. Wang et al. [40] demonstrated a change in an SNP belonging to a non-synonymous mutation in the sixth exon, resulting in a change in kernel size. Jiao et al. [41] demonstrated that a point mutation in *OsSPL14* perturbs OsmiR156-directed regulation of *OsSPL14*, generating an ‘ideal’ rice plant with a reduced tiller number, increased lodging resistance, and enhanced grain yield. Moreover, the association mutations may affect transcription themselves by increasing the affinity to activators of transcription factors to increase expansin gene abundance. Alternatively, they may affect modifications to DNA, which, in return, can affect transcription and other DNA-processing events, such as methylation. DNA methyl transferases (or methylases) recognize specific nucleotide sequences and methylate-specific nucleotides in those sequences at specific sites. Single-nucleotide mutations can either create or remove such sites [42]. Therefore, the identification of the *PgrEXP22* allele holds significant potential for investigating the interplay between seed weight genes, as the mutation in the *PgrEXP22* allele might alter the structure of the corresponding protein. Additionally, for a greater degree of genetic improvement, further candidate gene function analysis and research should be conducted for *PgrEXP22*. Furthermore, the seed weight-associated SNP identified in this study can serve as a valuable tool for facilitating the genetic improvement of seed weight in pomegranate-breeding programs through the utilization of marker-assisted selection techniques.

## 5. Conclusions

We performed a genome-scale analysis of the expansin gene family in pomegranate with a special emphasis on seed weight. A total of 29 pomegranate expansin genes were obtained. Our analysis has provided information for understanding the expansin superfamily, including gene evolution, gene structure, protein motifs, collinearity relationship, and gene expression patterns. Additionally, the association between *PgrEXPs* and seed weight in pomegranate showed that *PgrEXP22* is significantly associated with 100-seed weight and 100-sarcotesta weight. Our results are useful for improving the understanding of the impact of expansin on seed weight. In addition, the molecular marker of *PgrEXP22* may serve as a tool for the genetic improvement of seed weight in breeding programs of pomegranate.

## Figures and Tables

**Table 1:** Location, genotype, and alleles for the SNP/Indel in *PgrEXPA22* and *PgrEXPA6*.

Gene	SNP/Indel	Location	Genotypes and Alleles	Frequency of Genotypes and Alleles
*PgrEXPA22*	g.696T>C	exon	TT/TC/CC T/C	0.4158/0.3861/0.1980 0.6089/0.3911
	g.1004T>A	intron	TT/AT/AA T/A	0.4851/0.3366/0.1782 0.6535/0.3465
	g. 1088_1089insTGA	intron	INDEL+INDEL+/ INDEL+INDEL-/ INDEL-INDEL- INDEL+/INDEL-	0.5149/0.3168/0.1683 0.6733/0.3267
	g.1779T>G	UTR	TT/TG/GG T/G	0.5050/0.3267/0.1683 0.6684/0.3316
*PgrEXPA6*	g.1170A>G	intron	AA/AG/GG A/G	0.7129/0.1188/0.1683 0.7723/0.2277
	g.1237T>C	intron	TT/TC/CC T/C	0.7426/0.1089/0.1485 0.7971/0.2029
	g.1658C>G	intron	CC/CG/GG C/G	0.7129/0.1287/0.1584 0.77725/0.22275
	g.1745A>G	intron	AA/AG/GG A/G	0.7129/0.1287/0.1584 0.77725/0.22275
	g.2538C>G	exon	CC/CG/GG C/G	0.7129/0.1188/0.1683 0.7723/0.2277

**Table 2:** The association between polymorphic loci of PgrExps and seed weight traits of 101 pomegranate accessions.

SNP/Indel	Genotypes (Numbers)	100-Seed Weight (g)	100-Kernel Weight (g)	100-Sarcotesta Weight (g)	PSW (%)
g.696T>C	CC (20)	37.66 ± 6.43 b	3.99 ± 0.26	33.67 ± 6.54 b	89.14 ± 1.81 b
	TC (39)	40.12 ± 7.60 b	4.09 ± 0.50	36.03 ± 7.40 b	89.54 ± 1.73 b
	TT (42)	46.82 ± 12.29 a	4.11 ± 0.58	42.71 ± 12.08 a	90.69 ± 2.52 a
g.1004T>A	AA (18)	38.52 ± 7.04 b	4.03 ± 0.40	34.48 ± 7.07 b	89.25 ± 1.88
	AT (34)	41.01 ± 6.94 ab	4.08 ± 0.47	36.93 ± 6.76 ab	89.89 ± 1.42
	TT (49)	44.83 ± 12.57 a	4.10 ± 0.56	40.73 ± 12.38 a	90.22 ± 2.66
g.1088_1089insTGA	INDEL+INDEL- (32)	41.41 ± 6.97 ab	4.07 ± 0.48	37.33 ± 6.77 ab	90.00 ± 1.38
	INDEL+INDEL+ (52)	44.55 ± 12.32 a	4.11 ± 0.57	40.44 ± 12.12 a	90.18 ± 2.59
	INDEL-INDEL- (17)	37.82 ± 6.97 b	4.01 ± 0.27	33.81 ± 7.10 b	89.08 ± 1.97
g.1779T>G	GG (17)	38.08 ± 6.90 b	3.99 ± 0.26	34.09 ± 7.03 b	89.21 ± 1.92
	TT (51)	44.73 ± 12.37 a	4.11 ± 0.58	40.62 ± 12.17 a	90.20 ± 2.61
	TG (33)	41.08 ± 7.04 ab	4.08 ± 0.48	37.01 ± 6.85 ab	89.90 ± 1.44
g.1170A>G	AA (72)	42.86 ± 9.82	4.09 ± 0.49	38.77 ± 9.66	90.07 ± 2.14
	AG (12)	42.99 ± 11.93	4.04 ± 0.43	38.95 ± 11.77	90.10 ± 2.22
	GG (17)	40.15 ± 11.54	4.08 ± 0.61	36.07 ± 11.37	89.27 ± 2.40
g.1237T>C	CC (15)	41.41 ± 11.72	4.16 ± 0.60	37.25 ± 11.60	89.36 ± 2.55
	TC (11)	41.73 ± 11.64	3.98 ± 0.39	37.75 ± 11.54	89.95 ± 2.26
	TT (75)	42.72 ± 9.96	4.08 ± 0.50	38.64 ± 9.78	90.05 ± 2.12
g.1658C>G	CC (72)	42.86 ± 9.82	4.09 ± 0.49	38.78 ± 9.66	90.07 ± 2.14
	CG (13)	44.71 ± 12.99	4.06 ± 0.41	40.65 ± 12.83	90.36 ± 2.32
	GG (16)	38.58 ± 9.86	4.07 ± 0.63	34.51 ± 9.68	89.01 ± 2.21
g.1745A>G	AA (72)	42.86 ± 9.82	4.09 ± 0.49	38.77 ± 9.66	90.07 ± 2.14
	AG (13)	44.71 ± 12.99	4.06 ± 0.41	40.65±12.83	90.36 ± 2.32
	GG (16)	38.58 ± 9.86	4.07 ± 0.63	34.51 ± 9.68	90.07 ± 2.14
g.2538C>G	CC (72)	42.86 ± 9.82	4.09 ± 0.49	38.77 ± 9.66	90.07 ± 2.14
	CG (12)	42.99 ± 11.93	4.04 ± 0.43	38.95 ± 11.77	90.10 ± 2.22
	GG (17)	40.15 ± 11.54	4.08 ± 0.61	36.07 ± 11.37	89.27 ± 2.40

One-way analysis of variance (ANOVA) tests were used to test the difference between the different genotypes. Significant differences are indicated by different lowercase letters following the same column number at the same position (*p* < 0. 05).

## Data Availability

The original contributions presented in the study are included in the article/Supplementary Materials, further inquiries can be directed to the corresponding author/s.

## Supplementary Materials

The following supporting information can be downloaded at: https://www.mdpi.com/article/10.3390/genes15020212/s1, Supplementary File S1. Details on pomegranate accessions. Supplementary File S2. Primer sequences of two expansin genes. Supplementary File S3. Description of pomegranate expansin family genes. Supplementary File S4. Multiple sequence alignment of 29 PgrEXP proteins.
