# Supplementary material for: Identification of Candidate Expansin Genes Associated with Seed Weight in Pomegranate (Punica granatum L.)
Source: Genes (Basel). 2024 Feb 6;15(2):212. doi: 10.3390/genes15020212 (PMC10888256; doi:10.3390/genes15020212)

Figure S1. Multiple sequence alignment of 29 PgrEXP proteins. The signal peptide, conserved domain, conserved amino acid sites, and EXLA extension are clearly marked with black lines or arrows.

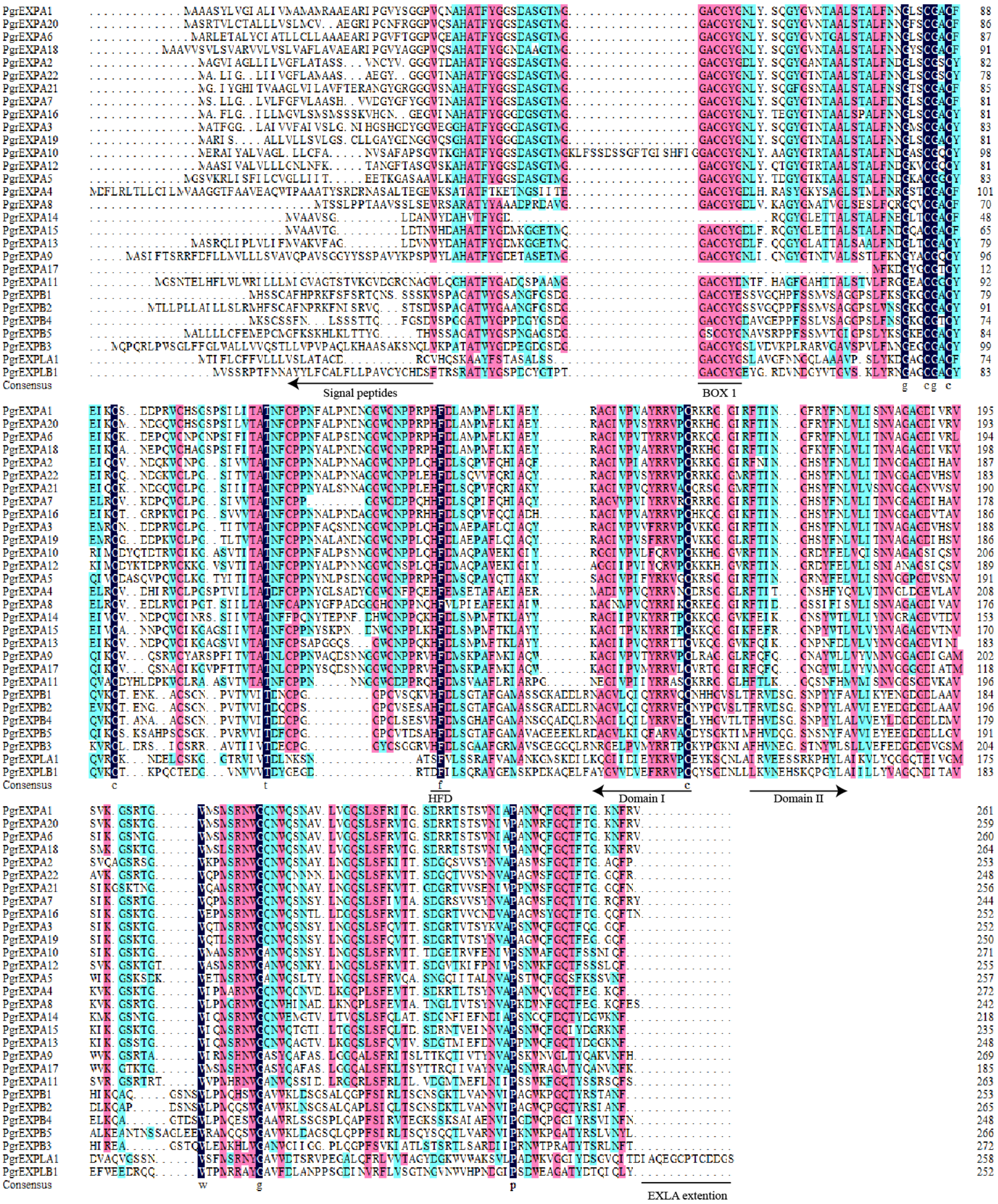

Supplement: Supplementary file 1 [file Supplementary File S4.pdf › Supplementary File S4.pdf]
